# Heart Dissemination: A Clinical Case of Melanoma

**DOI:** 10.1155/2021/8562402

**Published:** 2021-06-30

**Authors:** Diogo André, Teresa André, Fabiana Gouveia, Rafael Nascimento, António Chaves, Maria Braza˜o

**Affiliations:** ^1^Internal Medicine, Hospital Central do Funchal, Funchal, Portugal; ^2^Oncology, Hospital Central do Funchal, Funchal, Portugal

## Abstract

*Introduction*. Primitive malignant heart tumours are rare, specific cases. The presence of cardiac metastases, often in the pericardium, besides indicating disseminated oncological disease, represents a diagnostic challenge since they tend to be asymptomatic. Malignant cutaneous melanoma (MCM) is the neoplasm that most often affects the heart. *Patients and Methods*. The authors describe a case report of a 59-year-old female patient with a history of non-insulin-treated diabetes mellitus, arterial hypertension, dyslipidemia, and remitting cutaneous malignant melanoma who underwent skin excision, lymphadenectomy, and adjuvant chemotherapy in 1996. In April 2014, she resorted to emergency service due to epigastric pain and progressive tiredness. Due to the persistence of the complaints, abdominal ultrasound was performed, which showed a large pericardial effusion, corroborated later by teleradiography and echocardiography. The patient underwent pericardiocentesis, which isolated neoplastic cells. A computed tomography study of the chest, abdomen, and pelvis revealed bilateral and pericardial pleural effusion, as well as alterations suggestive of pericardial and pulmonary metastasis. Later, fine-needle aspiration puncture of the left posterior cervical nodule confirmed histologically malignant melanoma metastasis. *Discussion*. Given the natural history of melanoma that when metastasized has an overall survival of 15–20% for 5 years, its metastatic spread may occur several years after its surgical excision. Thus, patients with a history of melanoma and heart failure who develop new cardiac symptoms of unknown aetiology should undergo imaging studies such as echocardiography, computed tomography, and magnetic resonance imaging.

## 1. Introduction

Primary cardiac tumours are specific cases, and in adults, the most frequent malignant tumours are angiosarcomas, rhabdomyosarcomas, and fibrosarcomas [1–3]. Regarding benign neoplasms, myxomas and lipomas are the most prevalent [1].

The heart and pericardium are often affected in the context of disseminated metastatic tumour [1, 4–6]. Secondary cardiac metastasis is found in 20% of autopsies and approximately 10% of metastatic cancer patients [1, 4–6]. Any cardiac structure may be metastasized, but the epicardium is the preferred site, as described in 75.5% of the cases, and at least half of these are accompanied by pericardial effusion [1, 7]. The main forms of neoplastic dissemination are essentially by lymphatic route, hematogenous route, and contiguous extension [1, 7].

The main malignancies with frequent cardiac involvement are the lung and breast cancers, mainly due to their anatomical proximity and high prevalence [1, 2, 6, 7]. Nonepithelial tumours have cardiac involvement in 22.7% of cases [2]. Lymphomas, in the context of secondary immunodeficiency, also present an extensive cardiac involvement [2].

Cardiac metastases indicate a state of advanced oncological disease, which usually are asymptomatic [4, 5]. In some cases, they may manifest as arrhythmias, acute coronary syndromes, pericardial effusion, and acute heart failure [1, 3–5].

Cardiac tamponade as the first manifestation of neoplasia is unlikely since, in addition to pericardial infiltration by malignant cells, the presence of other circumstances, such as an acute event or even lymphatic obstruction, is necessary [4, 6].

## 2. Case Presentation

A 59-year-old female patient presented with a personal history of non-insulin-treated diabetes mellitus, dyslipidemia, and hypertension, usually medicated with metformin, perindopril, and simvastatin.

The patient attended to emergency service (ES) due to epigastric pain and feeling of fullness, for which was submitted to dioctyl sulfosuccinate enema and later discharged. Due to the persistence of the complaints and, now, with progressive tiredness, the patient frequented the ES two days later, where she underwent an abdominal ultrasound, which revealed pericardial effusion.

Objectively, on admission, the patient was conscious, oriented, collaborative, eupneic at rest and hemodynamically stable with rhythmic heart tones, no murmurs, decreased vesicular murmur in the right pulmonary base, free and defenceless abdomen, and lower limbs without oedema.

The patient displayed on electrocardiogram a sinus rhythm, 90 bpm heart rate, low amplitude QRS complexes, and nonspecific alterations in ventricular repolarization; chest X-ray was performed with increased cardiothoracic index and bilateral pleural effusion, especially at the base of the right hemithorax; echocardiogram was with large pericardial effusion “swinging heart” and partial collapse of the right heart chambers. The patient is admitted to the cardiac intensive care unit, in the context of cardiac tamponade.

**Figure 1:**
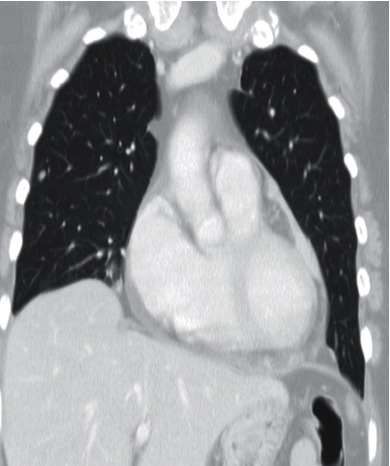
Thoracic computed tomography showing the presence of pericardial effusion.

During hospitalization, the patient underwent: pericardiocentesis, with a total drainage of 1250 cc, whose fluid, according to the pathological anatomy, evidenced neoplastic cells. She also underwent a thoracic-abdominal-pelvic computed tomography which revealed bilateral pleural effusion, with adjacent parenchymal atelectasis, slight pericardial effusion (Figure 1), and zones on the left side, with clear pericardial thickening suggestive of pericardial metastasis, after intravenous contrast. Several nodules in all lung lobes were indicative of metastases. The patient also underwent fine-needle aspiration biopsy of a cervical nodule, which has shown to be a melanoma metastasis. Later on, it was found out that the patient had malignant melanoma with inguinal ganglion metastasis in 1995 and completed chemotherapy, cutaneous excision, and lymphadenectomy in 1996.

During hospitalization, the patient had several periods of paroxysmal atrial fibrillation, despite chemical cardioversion with amiodarone. The patient was discharged with oral anticoagulation and digoxin, as an effective heart rate management agent.

## 3. Discussion

Melanoma most often affects the heart [5]. Its cardiac involvement is identified in more than 50% of autopsies, but it is challenging to diagnose since it spreads asymptomatically to the heart [1, 4, 7]. It is even more difficult to establish the prime diagnosis of melanoma through cardiac metastasis [6]. Once metastasized, the survival rate of patients with stage IV melanoma is very low as their 5-year survival is 15 to 20% [4, 7, 8]. It is metastasized via the hematogenous pathway, by the coronary arteries or the vena cava [1, 7]. Myocardial and pericardial involvement implies extracardiac metastatic involvement [1, 2, 7]. Like cardiac tamponade, the clinical presentation of metastatic melanoma through cardiac arrhythmia is rare. To date, the arrhythmogenic mechanism of melanoma is unknown. It is admitted that there is intramyocardial invasion with compromise of the right atrium impairing the electrical myocardial conduction system. The maintenance of paroxysmal atrial fibrillation presented in case may be explained by the electrical myocardial alterations due to melanoma [9].

Pericardiocentesis besides being lifesaving in the context of cardiac tamponade also allows establishing the etiological diagnosis. Treatment in these scenarios is limited to serous sclerosis or the establishment of pericardial windows [8].

New therapeutic regimens for malignant cutaneous melanoma (MCM)—immunotherapy, BRAF, and MEK inhibitors—demonstrated a significant improvement in relapse-free survival, although they are associated with cardiotoxicity. Among the documented adverse effects, the prolongation of QTc implies a monthly electrocardiographic evaluation [10].

The authors intend to emphasize that given the natural and unpredictable history of melanoma, the patient's history must be taken into account. Cardiac events can occur several years after surgical excision of skin melanoma [2, 5].

Due to the abovementioned conditions, all patients with the diagnosis of MCM should be followed and treated by a multidisciplinary team with Oncologists, Cardiologists, Radiologists, Endocrinologists, Surgeons, and Internal Medicine specialists, for proper treatment of the disease and adverse events from its' treatment.

## Data Availability

The data used to support the findings of this study have been deposited in the PubMed repository (DOI: https://doi.org/10.25504/FAIRsharing.a5sv8m).
